# Supplementary material for: The genome sequence of a seed weevil, Oxystoma pomonae (Fabricius, 1798) (Coleoptera: Apionidae)
Source: Wellcome Open Res. 2025 Nov 20;10:640. [Version 1] doi: 10.12688/wellcomeopenres.25092.1 (PMC12759273; doi:10.12688/wellcomeopenres.25092.1)
Supplement: Peer review history

# Reviewer report: Reviewer response for version 1

**DOI:** 10.21956/wellcomeopenres.27662.r140491

**Published:** 2026-01-02

**Authors:** Arun Arumugaperumal

**Affiliations:** 1 Department of Biotechnology, Rajalakshmi Engineering College, Thandalam, Chennai, Tamil Nadu, 602105, India

The data note describes the genome sequencing of a seed weevil, *Oxystoma pomonae*. Although it is a seed weevil it does not bring about much damage to crops. A female specimen was used for sequencing and the authors have identified a single X chromosome. The sequencing technology used aligned with the standard procedures of Darwin Tree of Life project sequencing protocol. A combination of long read sequencing and Hi-C sequencing were used to get high quality genome assembly. The assembly reported here is of size 1174.34 Mb spread among 11 chromosome molecules. The quality is evident from the high contig N50 value of 11.7 Mb. After scaffolding the N50 value has gone up to 115.24 Mb. Through Ensembl the annotation was completed and 14,352 genes were identified. BUSCO analysis with reference to endopterygota_odb10 dataset shows that the genome sequence reported is 98.2% complete. The contamination of sequences has been checked by using a GC% blob plot. This genome sequence will be an useful resource for comparison when the genome sequences of other members of Oxystoma are made available.

 Figure 2 caption can be changed to '....based on unassembled sequencing reads of *Oxystoma pomonae*'. The article can be indexed.

Are sufficient details of methods and materials provided to allow replication by others?

Yes

Is the rationale for creating the dataset(s) clearly described?

Yes

Are the datasets clearly presented in a useable and accessible format?

Yes

Are the protocols appropriate and is the work technically sound?

Yes

Reviewer Expertise:

Bioinformatics; Genomics

I confirm that I have read this submission and believe that I have an appropriate level of expertise to confirm that it is of an acceptable scientific standard.

# Reviewer report: Reviewer response for version 1

**DOI:** 10.21956/wellcomeopenres.27662.r140488

**Published:** 2025-12-26

**Authors:** Josephine R Paris

**Affiliations:** 1 University of Exeter, Devon, UK

This Data Note describes the genome assembly and annotation of the seed weevil (Oxystoma pomonae). The Background section on the species covers a good amount of information regarding its biology. The protocols, methods and materials are technically sound and all contain sufficient information for replication. The genome assembly data and RNAseq data are accessible via the ENA project accession numbers listed in the manuscript and in Table 1. I have no specific comments that need to be addressed.

Are sufficient details of methods and materials provided to allow replication by others?

Yes

Is the rationale for creating the dataset(s) clearly described?

Yes

Are the datasets clearly presented in a useable and accessible format?

Yes

Are the protocols appropriate and is the work technically sound?

Yes

Reviewer Expertise:

population genomics of non-models

I confirm that I have read this submission and believe that I have an appropriate level of expertise to confirm that it is of an acceptable scientific standard.
